# Phytochemical Screening and* In Vivo* Antimalarial Activity of Two Traditionally Used Medicinal Plants of Afar Region, Ethiopia, against* Plasmodium berghei* in Swiss Albino Mice

**DOI:** 10.1155/2019/4519298

**Published:** 2019-06-02

**Authors:** Sibhatu Gebrehiwot, Mohammed Shumbahri, Amelework Eyado, Tilahun Yohannes

**Affiliations:** ^1^Department of Biology, Raya University, Maichew, Tigray, Ethiopia; ^2^Department of Biology, Samara University, Samara, Afar, Ethiopia; ^3^Department of Biomedical Sciences, Addis Ababa University, Addis Ababa, Ethiopia; ^4^Department of Biology, University of Gondar, Gondar, Ethiopia

## Abstract

The objective of the present study was to investigate phytochemical components, antiplasmodial activity (*in vivo*) and evaluate the toxicity of two local medicinal plants, namely,* Salvadora persica* L. and* Balanites rotundifolia* (Van Tiegh.) used in Afar ethnomedicine for the treatment of malaria. In this study, phytochemical screening has been done using standard methods and the existence of antiplasmodial compounds was detected in these plant extracts. Four-day Peter's test was used to determine parasite inhibition, PCV was determined by Wintrob's method, and effects against loss of body weight and improvements on survival time were determined. LD50s of the crude extracts have been also done. Acute toxicity studies of the extracts were carried out in Swiss albino mice prior to antimalarial activity test. All extracts revealed no obvious acute toxicities on mice up to the highest (5000mg/kg) dose given. The crude extract was estimated to have oral median lethal dose higher than 5,000 mg/kg. With the 4-day suppressive test, both plant extracts demonstrated dose-dependent significant reduction in parasitemia level at all test doses compared to the negative control: in the extract of* B. rotundifolia* 500 mg/kg extract (60.59±3.25%), 350 mg/kg extract (48.1±1.4), and 200 mg/kg extract (41.33±1.1%) were found. And in case of* S. Persica* 500 mg/kg extract (50.6±4.01%), 350 mg/kg extract (35.85±0.89), and 200 mg/kg extract (27.69±1.14%) were found. The results of this study provide support for the traditional therapeutic value and the reported antimalarial activity.

## 1. Introduction

Malaria is one of the major diseases in developing countries causing an estimation of 219 million cases of malaria occurring worldwide in 2017 [1]. According to WHO (2015) report, the huge majority of deaths (99%) were found to be due to* Plasmodium falciparum* malaria [2, 3]. The largest burden of malaria morbidity is in Africa, with 200 million cases (92%) in 2017 [1].Malaria exists in all parts of Ethiopia, except in the central highlands, and 56 million people are at risk [4]. Afar Region is characterized by the lowland areas (≤1500 m altitude) with hot or warm climate. This special geography and climate leads to malaria outbreak epidemics and high prevalence of malaria disease in Afar region [5]. Ethiopia is a home of diversified flora which have been widely used as traditional medicine. Nevertheless, only a few of them are scientifically studied as source of drugs. The researches made so far on Ethiopian medicinal plants have been engaged mostly in producing inventories and checklists; only very few have been touched up by modern research where their principal component has been analyzed and defined [6, 7]. Apparently, lack of scientific proof of efficacies claimed by traditional medical practitioners in Ethiopia in general and in Afar region in particular has led us to design this study. The present research therefore aimed at investigating phytochemical screening, antiplasmodial activity (*in vivo*) and evaluate the toxicity of two local medicinal plants, namely,* Salvadora persica and Balanites rotundifolia *used in Afar ethnomedicine for the treatment of malaria.

## 2. Materials and Methods

### 2.1. Plant Materials Collection

The plant samples were collected from their natural habitat, the river forest of Aba'ala District, Afar region Ethiopia. The selection of plants was done on the basis of traditional reputation of particular plants for efficacy in the treatment and management of malaria as used by traditional health practitioners (THP) and the local communities. The plants were identified and authenticated by a Botanist, at Samara University, and the voucher specimens were deposited in the National Herbarium of Addis Ababa University. The medicinal plants used in this experiment were roots and leaves* Salvadora persica *and* Balanites rotundifolia, *respectively.

### 2.2. Experimental Methods

#### 2.2.1. Preparation of Crude Plant Extracts

Plant parts were openly dried at room temperature. The dried parts were grounded into fine powder. The macerated samples either in aqueous or organic solvent were rotated on a shaker for 24 hours at room temperature. Then, each sample was filtered out using a Whatman No. 1 filter paper. The filtered aqueous extracts were concentrated by lyophilization or freeze-drying. The filtered organic extracts were dried using a rotavapor at 40°C. The extracts were stored at -20°C until being used [8].

### 2.3. *In Vivo* Acute Toxicity Study of the Crude Plant Extracts

The crude extracts of the root and leaves of the two claimed medicinal plants, proposed for the antimalarial test against* P. berghei*, were assessed for their toxicity in noninfected male Swiss albino mice aged 6-8 weeks and weighing 25-35 grams according to the standard guideline of Organization for Economic Cooperation and Development (OECD) [9]. A total of 20 mice were selected, for the test of each plant extract and randomly divided into four groups of five mice per cage: one control group and three test groups. All mice were fasted overnight for 4 h before and 2 h after the administration of the extract [10]. And 0.2ml of methanol and/or chloroform extracts of the selected medicinal plants were given orally in an increasing dose, 2000, 3000, and 5000mg/kg for the mice in group one, two, and three, respectively, for four days. The mice in the control group received 0.2 ml of respective vehicle of each extract (3% Tween 80). Then, the mice were observed continuously for 1 hour, intermittently for 4 hours, and a period of 24 hours for gross behavioral changes such as feeding, lacrimation, mortality, and other signs of acute toxicity manifestations; this was done as per the standard toxicity study guideline OECD [9].

### 2.4. *In vivo* Evaluation of the Antimalarial Activity of Plant Extracts

#### 2.4.1. Suppressive Test

In studying the antiplasmodial activities of the plant extracts, the standard four-day suppressive method was employed [11]. Male Swiss albino mice weighing 25-35 were infected with 10^6^* P. berghei* and randomly divided into five groups of five mice per cage. The infected mice were randomly divided into three test groups and two controlled groups (each for Chloroquine as a standard drug and dH_2_O or 3% Tween 80 as a negative control). The test extracts were prepared in three different doses after toxicity test was done and Chloroquine at 25 mg/kg in a volume of 0.2 ml and vehicles at 0.5 ml/mouse. Each extract was administered as a single dose per day. All the extracts and the drug were given through intragastric route by using standard intragastric tube to ensure safe ingestion of the extracts and the drug [12]. Treatment was given after 3 hours of infection on day 0 and was continued daily for four days (i.e., from day 0 to day 3). On the fifth day (D4) blood samples were collected from tail snip of each mouse [12, 13]. Thin smears were prepared and stained with Giemsa solution. Then, each stained slide was examined under the microscope with an oil immersion objective of 100x magnification power to evaluate the percent suppression of each extract with respect to the control groups. The parasitaemia level was determined by counting the number of parasitized erythrocytes out of 100 erythrocytes in random field of the microscope. To make the data more reliable the parasite count was also performed by an experienced technician (double blinded) [8]. Percent of parasitaemia and percentage of suppression were calculated using the following formulas [14, 15].

(1) % Parasitaemia=No.  of  parasitized  RBCTotal  number  of  RBC  counted×100% Suppression=Parasitaemia  in  negative  control−parasitaemia  in  treated  groupparasitaemia  in  negative  control×100

#### 2.4.2. Determination of Packed Cell Volume

The packed cell volume (PCV) of each mouse was measured before infection and on day 4 after infection. For this purpose, blood was collected from tail of each mouse in heparinized microhematocrit capillary tubes up to 3/4th of their length. The tubes were sealed by crystal seal and placed in a microhematocrit centrifuge (Hettich haematokrit) with the sealed ends out wards. The blood was centrifuged at 12,000 rpm for 5 minutes. PCV was measured to predict the effectiveness of the test extracts in preventing hemolysis resulting from increasing parasitemia associated with malaria, using Wintrobe method [15, 16].

(2) PCV=volume  of  erythrocytes  in  a  given  volume  of  bloodtotal  blood  volume×100

#### 2.4.3. Determination of Mean Survival Time

Mortality was monitored daily and the number of days from the time of inoculation of the parasite up to death was recorded for each mouse in the treatment and control groups throughout the follow-up period. The mean survival time (MST) for each group was calculated using the following formula [15].

(3) MST=Sum  of  survival  time days of  all  mice  in  a  groupTotal  number  of  mice  in  that  group

#### 2.4.4. Determination of Body Weight Change

The body weight of each mouse in all the groups was measured before infection (day 0) and on day-4 in case of treatment; in the same fashion in case of subacute toxicity, it was measured before and after the different doses were given by a sensitive digital weighing balance (Scientech balance) [10].

### 2.5. Phytochemical Screening

The phytochemical screening of the two plants extract was carried out by following methods used by Trease and Evans [17] and Santaram and Harborne [18] to detect the presence or absence of certain bioactive compounds.

### 2.6. Data Analysis

Values were expressed as the mean +/- standard error of mean (SEM). Comparison of parasitaemia and statistical significance was determined by one way ANOVA followed by Scheffe's post hoc test using SPSS for window statistical package. Level of significance was taken at P<0.05.

### 2.7. Ethical Clearance

The animals were handled according to the international guidelines for the use and maintenance of experimental animals [19].

## 3. Results

### 3.1. Phytochemical Screening

The result of general phytochemical screening of powdered plant materials of* Salvadora persica* and* Balanites rotundifolia* showed the presence of many secondary metabolites. Phytoconstituents detected in plant samples such as sterols, triterpenes, alkaloids, saponins, quinone, steroidal glycosides, and resins were shown (Table 1).

### 3.2. Acute Oral Toxicity

With the acute toxicity test at the limit test dose of 5,000 mg/kg, neither mortality nor changes related to behavioral, neurological, and physical profile were observed within the first 24 h and during the 14 days' follow-up period.

### 3.3. *In vivo *Evaluation of the Antimalarial Activity of Plant Extracts

#### 3.3.1. Effect of Plant Extracts on Packed Cell Volume

Treatment with crude methanol extracts (200, 350 and 500mg/kg) of the leaf of* B. rotundifolia* did not prevent reduction in PCV but prevented body weight loss. The result showed a significant (P<0.05) reduction in PCV between days 0 and 4 in both negative controls and extract treated groups of mice, but there was no significant change in body weight (P>0.05)(Table 2).

Similarly, treatment with crude chloroform extracts (200mg/kg) of the leaf of* B. rotundifolia* did not prevent reduction in PCV due to parasitaemia but prevented body weight loss. The result showed a significant (P<0.05) reduction in both PCV and body weight between days 0 and 4 in both negative controls and extract treated groups of mice. Meanwhile, treatment with crude chloroform extracts (350 and 500mg/kg) prevented reduction in PCV and body weight loss due to parasitaemia. The result did not show a significant (P>0.05) reduction in both PCV and body weight between days 0 and 4 in extract treated groups of mice (Table 3).

On the other hand, treatment with crude methanol extracts (200, 350 and 500mg/kg) of the roots of* S. persica* prevented reduction in PCV and body weight loss due to parasitaemia. The result did not show a significant (P>0.05) reduction in PCV and body weight loss due to parasitaemia between days 0 and 4 in the extract treated groups of mice (Table 4).

Unlike the treatment with crude methanolic extracts, the treatment with crude chloroform extracts (200, 350 and 500mg/kg) of the roots of* S. persica* did not prevent reduction in PCV and body weight due to parasitaemia. The result showed a significant (P<0.05) reduction in PCV and body weight (except in the dose of 350mg/kg) between days 0 and 4 in both negative controls and extract treated groups of mice (Table 5).

#### 3.3.2. Effect of Crude Methanolic Plant Extracts on Parasitaemia and Mean Survival Time

The multiple comparison of antimalarial suppressive tests indicated that all the mice treated with the four extracts resulted in reduced parasite load as compared to their respective negative control groups. The extracts did not clear the parasite completely, whereas positive control groups treated with CQ phosphate, used as a standard antimalarial drug, at daily dose of 25mg/kg body weight totally cleared the parasite on day four under identical condition. Moreover, mice treated with the four extracts survived longer than mice in the corresponding negative control groups. Crude methanolic leaf extracts of* B. rotundifolia *produced a dose-dependent chemosuppressive effect at various doses employed. The crude methanolic leaf extracts of* B. rotundifolia* (200 mg; 3500 mg; 500 mg/kg) significantly (P<0.05) suppressed the parasitaemia. Furthermore, statistically), the mean survival time of mice treated at all doses was significantly (P>0.05) longer than the negative control (Table 6).

The methanol root extract of* S. persica* revealed significant (P<0.05) suppressive effect on parasitemia at the oral dose range of 200-500mg/kg per day compared to the untreated control. The average parasitemia of the mice treated with the highest dose (500mg/kg) was 35.6±3.14% and untreated control have 72.06±0.96%, which is by far greater than the treatment groups. The suppressive effect of the extract was also found to be dose dependent, which increased with increasing in the concentration of the extract, the highest (50.6±4.01%) being recorded at 500mg/kg dose. The methanol root extract of* S. persica* showed the highest antimalarial activity compared to the other extracts. The mice treated with the extracts had a survival time ranging from 9.4±0.24 to 10.4± 0.24 days, while the corresponding value of the untreated control group was 7.60±0.24 days. The mice treated with this extract survived significantly longer than mice in the negative control group. The mean survival time of the mice treated at the treated doses was relatively longer than the negative control. The extract showed significant effect on the mean survival time of the treatment groups compared to the untreated control, which increased as the dose increases (500mg/kg) (Table 7).

#### 3.3.3. Effect of Crude Chloroform Plant Extracts on Parasitaemia and Mean Survival Time

The crude chloroform leaf extracts of* B. rotundifolia *produced a dose-dependent chemosuppressive effect at various doses employed. The crude methanolic leaf extracts of* B. rotundifolia* (200 mg; 3500 mg; 500 mg/kg) significantly (P<0.05) suppressed the parasitaemia. Moreover, though statistically not significant (P>0.05), the mean survival time of mice treated at all doses was relatively longer than the negative control (Table 8).

Early malaria infection or Peters four days chemosuppressive activity test for the chloroform root extracts of* S. persica *produced a dose-dependent chemosuppression activity. The highest suppression of parasitaemia was observed at the dose of 500mg/kg body weight of mice. Percentage suppression was observed to increase as extract concentration increased. After four days treatment with the different extract doses, the mean parasitaemia of the test groups ranged from 58.74±0.79 to 43.6±2.02 while the corresponding value of the negative control group was 75.92±1.09%. The mice treated with CQ were completely free from the parasites on day four. The antimalarial activity produced by the extract was statistically significant (P<0.05) when related to negative control (Table 9).

## 4. Discussion

The phytochemical screening of extracts of* S. persica *and* B. rotundifolia *revealed the presence of different classes of secondary metabolites that have antiplasmodial activity in other plants [20], Tannins [21], alkaloids [22], and phenols [23]. The presence of these metabolites in* Salvadora persica *was also indicated in another study [24]. The antiplasmodial activity observed in many plants [25]and also in this study could have resulted from these metabolites which could be acting singly or in synergy with one another to exert the observed antimalarial activity. The antiplasmodial activity of extracts of* S. persica *and* B. rotundifolia* might also be attributed to the presence of alkaloids that have also been detected in both plants. Moreover, the antimalarial activities exhibited by these extracts may also be due to the presence of other active compounds such as phytosteroids and flavonoids, which are metabolites that have been proved to possess potential immunomodulatory effects in other plants [26] which as a consequence might have some impact on the host-parasite interrelationship. Herbal medicines are often regarded as safe because they are “natural”; but some products that contain bioactive principles have the potential to cause adverse effects [27]. To this end, toxicity, which is the main concern of indigenous therapeutic preparations [28], was addressed in the present study and the demonstrated lack of toxicity of the extracts in mice. The fact that changes in general behavior, effect on body weight and mortality, which are critical for the evaluation of adverse effects of a compound on test animals, were not evident on the test animals is good evidence for the absence of toxicity. This fulfills the criteria set for lack of acute toxicity by CDER [29]. Thus, since these plants are believed to have several traditional medicinal uses, including malaria treatment by different traditional healers in Afar region, the experimental determination of lack of acute toxicity would justify the use of the plant extracts for malaria treatment at primary health care level. Both the methanolic and chloroform extracts of* Salvadora persica *exhibited comparable suppressive activity on* P. berghei *which is in agreement with previous work on* in vitro* antimalarial activities [30]. Furthermore, from the present study both plant extracts exhibited promising suppressive activity on* P. berghei. *The highest suppression in both plant extracts was shown at the maximum dose given (500 mg/kg). This might be due to the fact that the active compounds, responsible for the antimalarial activity, mostly occur in low levels in natural products and activity may not be detected in lower doses [31]. It is interesting to note that the antimalarial activities observed in both plant extracts are from hydrophilic extracts as these extracts are closer in composition to the aqueous preparations commonly used by traditional practitioners [32]. PCV was measured to evaluate the effectiveness of the crude extracts in preventing hemolysis due to a rising parasitaemia level. In untreated mice, the parasite count increased and the hematocrit PCV decreased markedly from day to day till the death of mice; the same phenomenon was observed in other study [25]. In the present study there has been a strong association between the mean survival time and the suppression capacity of the plants which is in agreement with another study [33].

## 5. Conclusion

Antimalarial activities as well as the lack of toxicity of the extracts found in the present study may confirm the claim by traditional practitioners for the use of the plants against malaria and suggest their ethnopharmacological usefulness as antimalarials. Root extracts of* Salvadora persica* and leaf extracts* of Balanites rotundifolia* displayed significant chemosuppression and may serve as antimalarial treatment in primary health care where the novel drugs are inaccessible and unaffordable. It is suggested that further studies should be done to further elucidate and characterize possible approaches (herbal formulation) required in the use of these plants in the ethnomedical practices in primary health care.

## Figures and Tables

**Table 1:** Results of the phytochemical screening of the extracts of two plants traditionally used in Afar, Ethiopia.

Major tested phytoconstituents	Extracts	
	*B. rotundifolia*	*S. persica*

Phenol	-Ve	-Ve
Flavanoid	+Ve	-Ve
Tannin	-Ve	-Ve
Steroids	-Ve	+Ve
Triterpenoids	+Ve	+Ve
Steroidal glycosides	-Ve	+Ve
Alkaloids	+Ve	+Ve
Quinines	-Ve	+Ve
Saponins	+Ve	-Ve
Resins	-Ve	+Ve
Glycosides	+Ve	+Ve

+: secondary metabolite present.

-: secondary metabolite absent.

**Table 2:** Effect of crude methanolic leaf extracts of *B. rotundifolia *on body weight and PCV of *P. berghei* infected mice.

Dose mg/kg extract	PCV		Mean PCV	Body weight		Mean BWT
			% change			% change
	Day-0	Day-4		Day-0	Day-4	

*NC*	54.96±1.3	49.78±1.87	-9.55±4.2 ^a^	24.82±1.22	22.16.98±1.46	-11.03±1.4 ^a^
*200*	54.48±0.96	51.32±0.78	-5.83±1.19 ^a^	27.14±0.51	27.84±1.44	2.71±0.61 ^b^
*350*	52.38±2.01	48.76±3.16	-7.4±2.83 ^a^	27.94±0.41	29.3±6.9	4.38±0.87 ^b^
*500*	53.9±2.81	50.96±2.82	-5.51±0.96 ^a^	28.84±0.72	30.18±0.51	4.62±0.42 ^b^
*QC*	54.96±0.84	56.62±0.57	4.51±1.71 ^b^	31.4±0.68	33.5±2.5	6.79±1.3 ^b^

Means in a column followed by the same letter do not differ significantly (P>0.05).

*Key*= values are presented as M±SEM; n=5; CQ= Chloroquine Phosphate; NC= negative control (0.2ml of dH2O).

**Table 3:** Effect of crude chloroform leaf extracts of *Balanites rotundifolia *on body weight and PCV of *P. berghei* infected mice.

Dose mg/kg extract	PCV		Mean PCV	Body weight		Mean BWT
			% change			% change
	Day-0	Day-4		Day-0	Day-4	

*NC*	64.86±0.65	49.98±2.34	-22.78±4.3 ^a^	24.8±0.96	22.6.±1.07	-8.67±3.6 ^b^
*200*	62.18±0.96	48.18±3.38	-22.14±5.54 ^a^	27.2±0.58	26±0.37	-4.41±2.3 ^a^
*350*	60.02±1.1	52.82±2.51	- 7.68±5.67 ^b^	27 ±0.44	26±0.58	-3.47±0.87 ^a^
*500*	62.62±1.05	54.5±1.41	-12.82±3.04 ^b^	25±0.71	24.8±1.39	-0.8±1.08 ^a^
*QC*	64.91±0.64	61.74±0.59	-4.81±0.63 ^b^	26.6±1.36	26.4±0.8	-1.5±0.8 ^a^

Means in a column followed by the same letter do not differ significantly (P>0.05).

*Key*= values are presented as M±SEM; n=5; CQ= Chloroquine Phosphate; NC= negative control (0.2ml of dH2O); BWT=body weight.

**Table 4:** Effect of crude methanolic root extracts of *S. persica *on body weight and PCV of *P. berghei* infected mice.

Dose mg/kg extract	PCV		Mean PCV	Body weight		Mean BWT
	% change					% change
	Day-0	Day-4		Day-0	Day-4	

*NC*	55.05±1	45.01±2.06	-18.03±9.9 ^a^	29.46±0.66	27.58±0.43 ^a^	-6.39±0.79 ^a^
*200*	54.57±1.31	52.25±1.16	-3.76±0.75 ^b^	24.62±1.36	25.84±1.36 ^a^	4.91±1.19 ^b^
350	54.68±0.94	52.25±1.03	-4.76±0.67 ^b^	28.88±0.28	29.72±0.45 ^a^	2.9±0.1 ^b^
500	54.25±0.8	51.72±0.94	-4.67±0.73 ^b^	25.4±0.70	25.9±1 ^a^	3.81±1.7 ^b^
QC	54.85±0.38	58.06±0.83	4.58±0.61 ^b^	28.18±1	31.02±0.74 ^a^	10.31±2.3 ^b^

Means in a column followed by the same letter do not differ significantly (P>0.05).

*Key*= values are presented as M±SEM; n=5; CQ= Chloroquine Phosphate; NC= negative control (0.2ml of dH2O); BWT=body weight.

**Table 5:** Effect of crude chloroform leaf extracts of *S. persica* on body weight and PCV of *P. berghei* infected mice.

Dose mg/kg extract	PCV		Mean PCV	Body weight		Mean BWT
			% change			% change
	Day-0	Day-4		Day-0	Day-4	

*NC*	64.86±0.65	49.98±2.34	-22.78±4.3 ^a^	24.8±0.96	22.6.±1.07	-8.67±3.6 ^a^
*200*	60.92±0.97	52.54±2.06	-13.84±2.44 ^a^	25.6±0.58	23.3±0.66	-8.53±3.27 ^a^
*350*	61.36±1.35	54.56±0.97	-10.93±2.28 ^a^	26.2 ±0.86	25.2±0.66	-3.72±1.09 ^b^
*500*	61.74±0.72	48.62±1.62	-19.62±2.51 ^a^	25.8±0.66	23.4±0.51	-9.11±2.68 ^a^
*QC*	64.88±0.64	61.74±0.26	-4.81±0.63 ^b^	26.6±1.36	27.4±0.8	3.11±0.8 ^b^

Means in a column followed by the same letter do not differ significantly (P>0.05).

*Key*= values are presented as M±SEM; n=5; CQ= Chloroquine Phosphate; NC= negative control (0.2ml of dH2O); BWT=body weight.

**Table 6:** Effect of *B. rotundifolia* on percentage parasitemia, and survival time of *P. berghei* infected mice in the 4-day suppressive test.

Dose mg/kg extract	% Parasitaemia ± SEM	% Suppression	MST

*NC*	77.22±2.11	0.00	6±0.63
*200*	45.29±1.42	41.33±1.1	9±0.83 ^b^
*350*	40.08±2.81	48.1±1.4	10.2±0.2 ^b^
*500*	30.44±0.89	60.59±3.25	16.4±0.51 ^b^
*QC*	0.00	100	30(+) ^d^

Means in a column followed by the same letter do not differ significantly (P>0.05).

*Key*: values are presented as M±SEM; n=5; CQ= Chloroquine Phosphate; NC = negative control (0.2ml of dH2O); + = maximum days of followup; MST=mean survival time.

**Table 7:** Antimalarial activities of crude methanolic root extracts of *Salvadora persica *and mean survival time.

Dose mg/kg extract	% Parasitaemia ± SEM	% Suppression	MST

*NC*	72.06±0.96	0.00 ^a^	7.6±0.24 ^a^
*200*	52.21±0.57	27.69±1.14 ^b^	9.4 ±0.24 ^b^
*350*	46.22±0.93	35.85±0.89 ^b^	10.2±0.2 ^b^
*500*	35.6±3.14	50.6±4.01 ^c^	10.4±0.24 ^b^
*QC*	0.00	100 ^d^	30(+) ^c^

Means in a column followed by the same letter do not differ significantly (P>0.05).

*Key*: values are presented as M±SEM; n=5; CQ= Chloroquine Phosphate; NC = negative control (0.2ml of dH2O); + = maximum days of followup; MST=mean survival time.

**Table 8:** Antimalarial activities of crude Chloroform leaf extracts of *Balanites rotundifolia *and mean survival time.

Dose mg/kg extract	% Parasitaemia ± SEM	% Suppression	MST

*NC*	75.92±1.09	0.00	6±0.63 ^a^
*200*	56.48±0.9	25.6±1.72	8 ±0.83 ^a^
*350*	44.16±0.78	41.83±1.57	9.2±0.2 ^a^
*500*	33.42±2.77	55.98±3.17	9.4±0.51 ^a^
*QC*	0.00	100	30(+) ^b^

Means in a column followed by the same letter do not differ significantly (P>0.05).

*Key*: values are presented as M±SEM; n=5; CQ= Chloroquine Phosphate; NC=negative control (0.2ml of dH2O); += maximum days of followup; MST=mean survival time.

**Table 9:** Antimalarial activities of crude Chloroform root extracts of *S. persica *and mean survival time.

Dose mg/kg extract	% Parasitaemia ± SEM	% Suppression	MST

*NC*	75.92±1.09 ^a^	0.00 ^a^	6.4±2.44 ^a^
*200*	58.74±0.79 ^b^	22.62±0.59 ^b^	7.4 ±0.63 ^a^
*350*	55.04±0. 8 ^b^	27.89±1.26 ^b^	8.2±0.5 ^a^
*500*	43.6±2.02 ^b^	42.57±4.69 ^c^	8.4±0.6 ^a^
*QC*	0.00 ^c^	100 ^d^	30(+) ^b^

Means in a column followed by the same letter do not differ significantly (P>0.05).

*Key:* values are presented as M±SEM; n=5; CQ= Chloroquine Phosphate; PC=positive control; NC=negative control (0.2ml of 20% DMSO); += maximum days of followup; MST=mean survival time.

## Data Availability

The data used to support the findings of this study are included within the article.
